# IL-23 induces regulatory T cell plasticity with implications for inflammatory skin diseases

**DOI:** 10.1038/s41598-019-53240-z

**Published:** 2019-11-27

**Authors:** Arun K. Kannan, Zhi Su, Donna M. Gauvin, Stephanie E. Paulsboe, Ryan Duggan, Loren M. Lasko, Prisca Honore, Michael E. Kort, Steve P. McGaraughty, Victoria E. Scott, Stephen B. Gauld

**Affiliations:** 1 Abbvie Inc., 1 North Waukegan Road, North Chicago, IL 60064 USA; 2 Retired, Gurnee, IL USA

**Keywords:** Autoimmunity, Autoimmunity, Autoimmunity, Acute inflammation, Acute inflammation

## Abstract

Foxp3^+^ regulatory T cells (Tregs) represent a major fraction of skin resident T cells. Although normally protective, Tregs have been shown to produce pro-inflammatory cytokines in human diseases, including psoriasis. A significant hurdle in the Treg field has been the identification, or development, of model systems to study this Treg plasticity. To overcome this gap, we analyzed skin resident Tregs in a mouse model of IL-23 mediated psoriasiform dermatitis. Our results demonstrate that IL-23 drove the accumulation of Tregs; including a subpopulation that co-expressed RORγt and produced IL-17A. Genesis of this population was attenuated by a RORγt inverse agonist compound and clinically relevant therapeutics. *In vitro*, IL-23 drove the generation of CD4^+^Foxp3^+^RORγt^+^IL-17A^+^ cells from Treg cells. Collectively, our data shows that IL-23 drives Treg plasticity by inducing a population of CD4^+^Foxp3^+^RORγt^+^IL-17A^+^ cells that could play a role in the disease pathogenesis. Through this work, we define an *in vitro* system and a pre-clinical *in vivo* mouse model that can be used to further study Treg homeostasis and plasticity in the context of psoriasis.

## Introduction

Foxp3^+^ regulatory T cells (Tregs) are indispensable for the maintenance of tolerance to self and prevention of autoimmunity. The non-redundant role of Tregs in establishing immune homeostasis is exemplified by the development of multi-organ autoimmunity in humans and mice that harbor mutations in the Foxp3 locus^1,2^. Furthermore defects in the number, or function, of Tregs have been reported in an increasing number of autoimmune diseases^3^. These findings highlight the importance of characterizing the ontogeny, phenotype and function of Tregs, particularly in inflamed tissue. Such studies may help develop novel therapeutic strategies to treat a magnitude of autoimmune diseases.

While the majority of Tregs are derived from the thymus, they can also be induced from naive CD4^+^ T cells in tissue to mediate peripheral tolerance^4^. A number of tissue-specific Tregs have been described with distinct transcriptional profiles and specialized functions. These include Foxp3^+^ T cell subsets in the colonic lamina propria, visceral adipose tissue and the skeletal muscle^5^. In addition to this, an unusually high number of Foxp3^+^ T cells reside in the skin. This cutaneous Treg subset is a major component of both mice and human skin; they express distinct chemokine receptors and play a major role in the maintenance of immune homeostasis and prevention of autoimmunity in the skin^6,7^. Furthermore, a number of inflammation driven ‘effector’ Treg subsets have been described that are either protective or detrimental to the host depending on the inflammatory context and tissue^8^. From a protective standpoint, Treg subsets that express Tbet, GATA3/Irf4 or Bcl6 have been shown to be critical for the restraint of Th1^9^, Th2^10,11^ or Tfh^12^ responses, respectively. In addition, colonic RORγt^+^ Tregs play an important role in regulating Th17 cell responses and maintaining host-microbiome symbiosis in the intestinal compartment^13^. In stark contrast, under certain inflammatory conditions Tregs can be detrimental and have been shown to secrete effector cytokines and likely support immunopathology. Tregs that produce IFN-γ have been shown to exacerbate multiple sclerosis^14^ and type-1 diabetes^15^. Tregs that make IL-17A has been implicated in the pathogenesis of rheumatoid arthritis^16^ and psoriasis^17^. Psoriasis is a prototypic IL-17A and Th17 cell mediated autoimmune disease where targeting the inhibition of IL-23/IL-17 axis is a clinically validated approach for treatment^18^. In addition to an increase in RORγt^+^IL-17A^+^ Th17 cells, the psoriatic inflammatory cytokine milieu has been shown to drive production of IL-17A by Tregs^17^. While it’s clear that psoriatic lesional skin has a significant fraction of Foxp3^+^ cells that express IL-17A^7,17^, the ontogeny and the signaling nodes that regulate the generation and maintenance of these Th17-like Tregs (defined in the literature as Foxp3^+^IL-17A^+^ T cells) remains a significant gap in knowledge. Progress in this area has been hindered by a lack of models to study the development and function of Th17-like Tregs in systems relevant to human disease.

In the current study, IL-23 induced accumulation of Tregs (defined in this manuscript as CD4^+^Foxp3^+^ T cells) that show enhanced proliferative capacity in the skin. The increase in Treg cell numbers was associated with a significant increase in a population of Tregs that co-expressed RORγt and made IL-17A. The development and/or ability of these Th17-like Tregs (defined in this manuscript as CD4^+^Foxp3^+^RORγt^+^IL-17A^+^ T cells) to make IL-17A was regulated by RORγt signaling. The accumulation of this population was also reduced in the presence of clinically relevant therapeutics that neutralize TNF-α or IL-23. IL-23 by itself drove the generation of CD4^+^Foxp3^+^RORγt^+^IL-17A^+^ cells from isolated Tregs *in vitro*. Collectively, our data suggests that IL-23 drives the accumulation of Th17-like Tregs, a population that may be relevant to the pathogenesis of psoriasis.

## Results

### IL-23 induced inflammation drives accumulation of Tregs and IL-17A production by cutaneous Tregs

**Figure 1:**
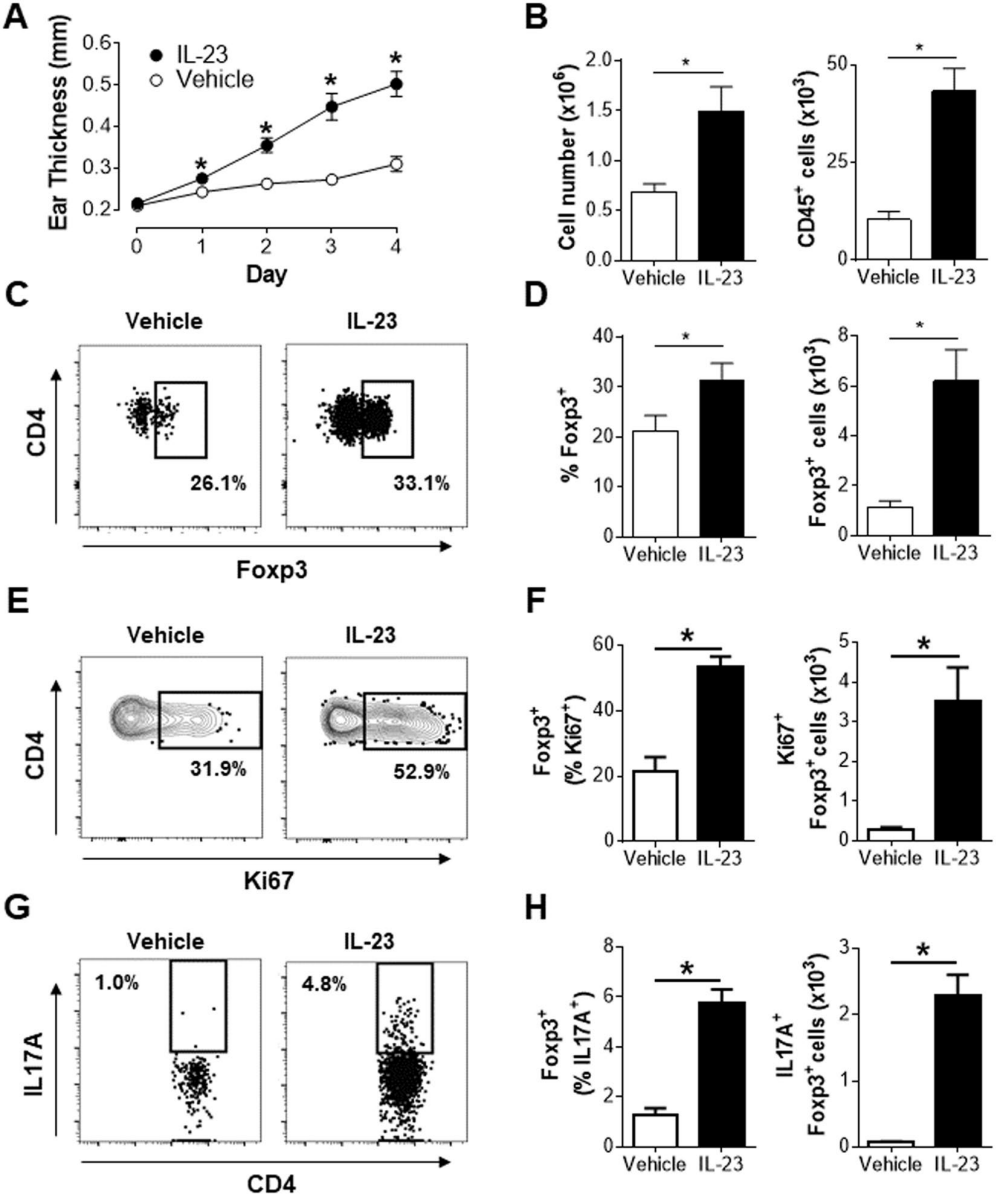
IL-23 induced inflammation drives accumulation of Tregs. Animals received four daily intradermal (ear) injections of vehicle or IL-23 (days 0–3) and were analyzed on day 4. (**A**) Measurement of ear thickness. (**B**) Total cellularity and number of CD45^+^ cells in the ear. (**C**) Representative flow cytometry plots of live CD45^+^CD4^+^TCRβ^+^ cells in the ear. (**D**) Frequency and number of Foxp3^+^ (live CD45^+^CD4^+^TCRβ^+^Foxp3^+^ cells) in the ear of mice. (**E** and **G**) Representative flow cytometry plots of Foxp3^+^ cells in the ear. (**F** and **H**) Frequency and number of the indicated cell populations in the ear. (**A**) Representative data of at least 3 independent experiments, n = 4. (**B**–**F**) Pooled data from two independent experiments, n = 8. (**G** and **H**) Data represents pooled analysis of 2 vehicle or IL-23 treated ears. Representative data from two independent experiment, at least n = 3 in each experiment. Live CD45^+^CD4^+^TCRβ^+^Foxp3^+^ cells in the ear are defined as Foxp3^+^ cells in the figure. *p < *0*.*05* determined by student’s t test.

Our understanding of the genesis and function of Th17-like Tregs in psoriasis is hindered by the fact that these cells are already differentiated in human skin. To overcome this hurdle, the plasticity of Tregs was investigated in a mouse model of psoriasiform dermatitis that is representative of human psoriasis^19,20^. As reported previously by our group^19^, IL-23 induced a robust, time dependent, increase in ear thickness (Fig. 1A) associated with significant increases in total cellularity and number of CD45^+^ leukocytes in the ear (Fig. 1B). IL-23 also significantly increased the cellularity of ear draining lymph nodes (Supplementary Fig. 1A). Previous studies have shown that Tregs are a major component of the CD4^+^ T cell compartment in the skin^6,7^. In agreement with these findings, ~20% of CD4^+^ T cells in the ear of vehicle treated animals were Tregs (Fig. 1C,D). Interestingly, IL-23 induced inflammation resulted in a significant increase in both the frequency and number of Treg cells in the ear (Fig. 1C,D) and draining lymph nodes (Supplementary Fig. 1B). Thus, in addition to the increase in thickness and total cellularity in the ear, IL-23 drove the accumulation of Tregs at the site of inflammation. Furthermore, IL-23 stimulated a significant fraction of Tregs to enter cell cycle based on the increased frequency and number of Ki67^+^ Tregs in the ear (Fig. 1E,F) and draining lymph nodes (Supplementary Fig. 1C). More importantly IL-23 induced a significant fraction of Tregs in both the skin and draining lymph nodes to make IL-17A. While only approximately 1% of ear and draining lymph node Tregs from vehicle treated animals expressed IL-17A, this was increased 5–6 fold upon IL-23 treatment (Fig. 1G,H; Supplementary Fig. 1D). In line with published reports on IL-23 driving IL-17A production by dermal αβ^+^ and γδ^+^ T cells^21–23^, IL-23 also induced IL-17A production in γδ^+^ T cells and Foxp3^−^αβ^+^ T cells (Supplementary Fig. 2A,B), this report will focus on the impact of IL-23 on Tregs. Collectively, these results suggest that IL-23 mediated inflammation supports the accumulation of a Treg population that co-expresses IL-17A. These findings are in alignment with the phenotype of Tregs found in the lesional skin of psoriatic patients^7^.

### IL-23 induces a population of Th17-like Tregs that is also sensitive to RORγt inhibition

**Figure 2:**
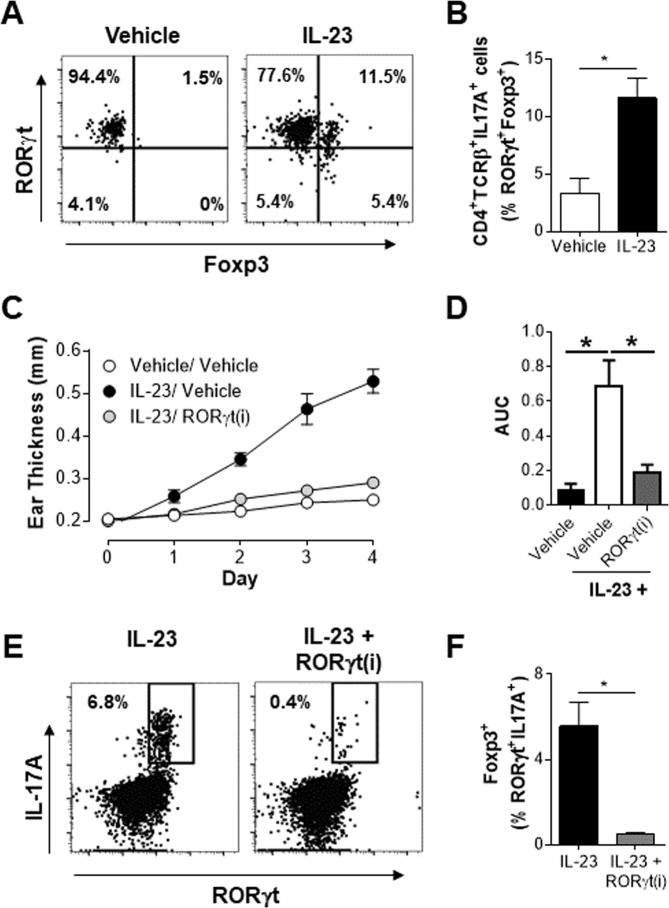
IL-23 induces a population of Th17 like Tregs that is also sensitive to RORγt inhibition. Vehicle or IL-23 treated ears were analyzed on day 4. (**A**) Representative flow cytometry plots of live CD45^+^CD4^+^TCRβ^+^IL-17A^+^ cells in the ear. (**B**) Frequency of live CD45^+^CD4^+^TCRβ^+^IL-17A^+^RORγt^+^Foxp3^+^cells in the ear. Data represents pooled analysis of 2 vehicle or IL-23 treated ears. Representative data from two independent experiments, at least n = 3 in each experiment. (**C**) Absolute ear thickness and (**D**) area under the curve (AUC) measurement in mice treated with vehicle or IL-23 in the presence or absence of a RORγt(i) at 100 mg/kg PO-QD. (**E**) Representative flow cytometry plots and (**F**) frequency of Th17-like Tregs cells in the draining lymph nodes of mice treated with IL-23 and either vehicle or 100 mg/kg of a RORγt(i) PO-QD. Live CD45^+^CD4^+^TCRβ^+^Foxp3^+^ cells in the ear and live CD4^+^TCRβ^+^Foxp3^+^ cells in the draining lymph nodes are defined as Foxp3^+^ cells in the figure. Data represents a study of n = 6 per group, with similar results on efficacy of RORγt(i) in IL-23 treated animals obtained from independent studies with other RORγt(i) compounds. *p < *0*.*05* determined by student’s t test.

Next, these cells were examined to determine if they shared other phenotypic features of Th17 cells. Characterization of IL-17A producing T cells (CD4^+^TCRβ^+^IL-17A^+^) in the ears of vehicle treated animals indicated that IL-17A^+^ cells are predominately RORγt^+^ and Foxp3^−^ (Fig. 2A). In contrast, in IL-23 treated animals a significant fraction (10–15%) of CD4^+^IL-17A^+^ cells co-expressed Foxp3 and RORγt (Fig. 2A,B). The sensitivity of these cells to pharmacological inhibition was assessed by use of a RORγt inverse agonist (RORγt(i)) that is known to significantly reduce IL-23 mediated skin inflammation (Stephen Gauld, *et al*., manuscript under review)^24^. Administration of an AbbVie RORγt(i) significantly reduced the IL-23 induced ear inflammation at all the time points analyzed (Fig. 2C) with an 84% reduction in the area under the curve measurement of ear thickness (Fig. 2D). Importantly, the accumulation of CD4^+^Foxp3^+^RORγt^+^IL-17A^+^ population in IL-23 treated mice was almost completely abrogated by RORγt inhibition (Fig. 2E,F). However, overall the reduction in disease severity observed in the presence of RORγt(i) is likely due a global effect on the reduction of IL-17A production by all RORγt^+^ subsets. These results clearly demonstrate that IL-23 supports the accumulation of a Treg cell population that also co-express functional RORγt and produce IL-17A. Similar to Th17 cells, the development and/or the effector function of this Th17-like Treg population is dependent on RORγt.

IL-23 driven Treg responses were further characterized to determine if IL-23 induced broad lineage instability of Tregs by inducing production of other effector cytokines. Th1-like Tregs have been shown to play a role in driving the pathogenesis of multiple sclerosis^14^ and type 1 diabetes^15^. Analysis of Tregs in the ear revealed that, while IL-23 induced a slight increase in the number of IFN-γ^+^ Treg cells (Supplementary Fig. 1E), IFN-γ^+^ Tregs were not enriched in the draining lymph nodes of IL-23 treated animals (Supplementary Fig. 1F). Thus the IL-23 mediated effects on Tregs were largely restricted to IL-17A and the IL-23-IL-17A axis.

### Clinically relevant therapeutics significantly impact the accumulation of Th17-like Tregs

**Figure 3:**
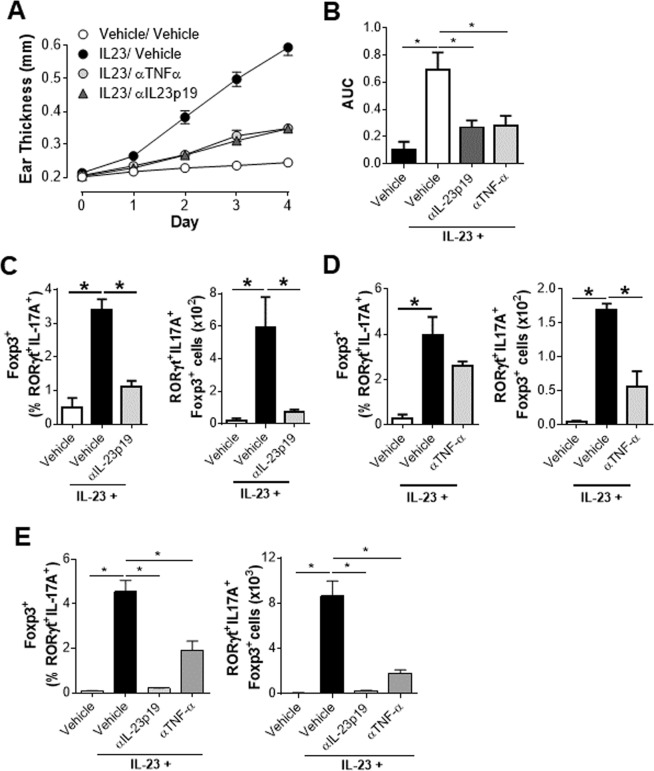
Clinically relevant therapeutics significantly impact the accumulation of Th17-like Tregs. All mice were analyzed on day 4. 2 hours prior to administration of vehicle or IL-23 (on day 0 and day 2), mice were treated (intraperitoneal injection) with vehicle, 15 mg/kg of anti-TNF-α or 15 mg/kg of anti-IL-23p19. (**A**) Absolute ear thickness and (**B**) area under the curve (AUC) measurement in mice treated with vehicle or IL-23 in the presence of vehicle, 15 mg/kg of anti-TNF-α or 15 mg/kg of anti-IL-23p19. (**C** and **D**) Frequency and number of Th17-like Tregs in the ear skin of mice treated with vehicle or IL-23 in the presence or absence of anti-IL-23p19 (C) or anti-TNF-α (D). Data represents pooled analysis of 2 ears for each data point, n = 4. *p < *0*.*05* using student’s t test. (**E**) Frequency and number of Th17-like Tregs in the draining lymph nodes. Data represents a study of n = 8 per group, with similar results on the efficacy of anti-TNF-α and anti-IL-23p19 in IL-23 treated animals obtained in a number of other independent studies. Live CD45^+^CD4^+^TCRβ^+^Foxp3^+^ cells in the ear and live CD4^+^TCRβ^+^Foxp3^+^ cells in the draining lymph nodes are defined as Foxp3^+^ cells in the figure. *p < *0*.*05* determined by student’s t test.

Inhibition of TNFα and IL-23 signaling nodes are clinically validated approaches for the treatment of psoriasis in patients^18^. The fact that Th17-like Tregs were enriched in inflamed skin led to the hypothesis that therapeutic agents that reduce disease severity will also reduce the accumulation of this hybrid population. To this end, IL-23 treated animals that also received antibodies against TNFα or the p19 subunit of IL-23 were analyzed. Both anti-TNFα and anti-IL23p19 showed robust efficacy in reducing IL-23 induced ear inflammation at all the time points analyzed (Fig. 3A) with anti-TNF-α and anti-IL23 p19 showing a 69% and 72% reduction in the area under the curve measurement of ear thickness respectively (Fig. 3B). Alongside changes in ear thickness, there was a dramatic reduction in the proportion and number of Th17-like Tregs in the ears of animals treated with anti-IL-23p19 (Fig. 3C). Accumulation of Th17-like Tregs in the ear was also significantly reduced by anti-TNFα treatment (Fig. 3D). IL-23 also induced a significant accumulation of Th17-like Tregs in the draining lymph nodes, and treatment with anti-IL-23p19 reduced this to basal levels (Fig. 3E). Interestingly, TNFα neutralization also resulted in a significant reduction in both the proportion and number of Th17-like Tregs in the draining lymph nodes (Fig. 3E). Thus, clinically relevant therapeutics that attenuate disease severity significantly reduced the accumulation of this Th17-like Treg population.

### Th17-like Tregs are preferentially generated from Tregs

**Figure 4:**
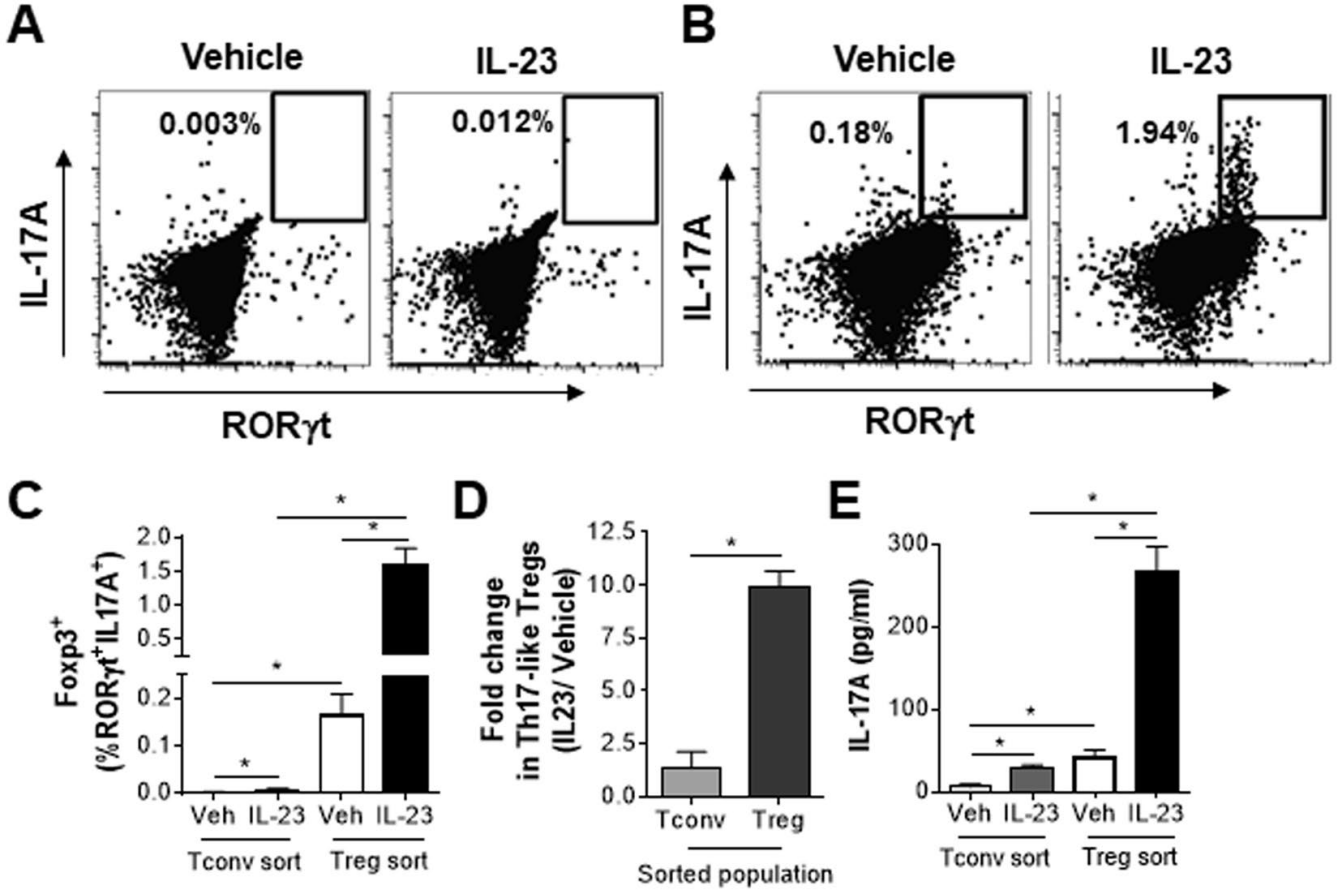
Th17-like Tregs are preferentially generated from Tregs. FACS sorted cells were treated with vehicle or IL-23 and stimulated with DynaBeads in the presence of anti-IFN-γ and anti-IL-4. On day 3, cells were re-stimulated with PMA and ionomycin in the presence of protein transport inhibitors prior to analysis. (**A** and **B**) Representative flow cytometry plots and (**C**) frequency of live Foxp3^+^RORγt^+^IL-17A^+^ cells in cultures of sorted CD4^+^GFP^−^ Tconv cells or CD4^+^GFP^+^ Tregs on day 3. (**D**) Fold change in generation of Th17-like Tregs from Tconv or Treg sorted cells, represented as ratio of IL-23 stimulation over vehicle control. (**E**) Sorted Tconv or Treg cells treated as above and supernatants were analyzed on day3 for the level of IL-17A. Data represents analysis of two independent experiments with cells sorted from pooled spleen and lymph node cells of 4 animals each time. *p < *0*.*05* determined by student’s t test.

It has been reported that Th17-like Tregs can be generated from either Foxp3^+^ Tregs or Foxp3^−^ Th17 cells depending on the inflammatory context^25^. To determine if IL-23 driven Th17-like Tregs could be generated from Treg or conventional T (Tconv) cells, GFP^−^ (Tconv) and GFP^+^ (Treg) CD4^+^ T cells were sorted from Foxp3-GFP reporter mice. After 3 days in culture, a fraction of sorted Tconv cells expressed Foxp3 (8–15%). However, IL-23 stimulation was only able to drive a very small fraction of these Foxp3^+^ cells to co-express RORγt and IL-17A (Fig. 4A,C). Interestingly, there was a robust increase in CD4^+^Foxp3^+^RORγt^+^IL-17A^+^ cells when sorted Treg cells were stimulated with IL-23 (Fig. 4B,C). Overall, when represented as a fold change in the IL-23 stimulated condition over vehicle control, it is clear that Th17-like Tregs are preferentially generated from Treg cells *in vitro* (Fig. 4D). More importantly, IL-23 stimulated sorted Tregs secreted significantly higher amounts of IL-17A protein compared to vehicle controls and sorted Tconv cells (Fig. 4E). IL-23 stimulated sorted Tregs also made higher levels of IL-10 compared to vehicle controls and sorted Tconv cells (Supplementary Fig. 3). Collectively, these data support the hypothesis that IL-23 induced Th17-like Tregs, that secrete IL-17A, are preferential generated from Treg precursor cells.

## Discussion

It is increasingly clear that there is considerable plasticity among T helper cell subsets and between Th17 and Treg cells in particular^26^. Recent studies have identified a number of effector like Treg populations that protect against, or drive, immune pathology depending on the site of inflammation and the inflammatory milieu^8^. For instance, depending on the inflammatory context, Tregs that share features of Th17 cells have been shown to regulate Th17 inflammation to maintain homeostasis in the gut^13^ and be protective in a mouse model of systemic lupus erythematosus^27^ or drive psoriatic inflammation^17,28^.

The ability to characterize the biology of Th17-like Tregs in psoriasis is hindered by the need for human tissue, the heterogeneity of human disease and the fact that these cells are already differentiated in the tissue. As a result, we have been unable to characterize the origin and signaling nodes regulating the generation and/or maintenance of Th17-like Tregs. In an effort to better understand these cells and the role of Tregs in inflammatory skin diseases, the homeostasis and function of Tregs during psoriasis-like skin inflammation was investigated in an intradermal IL-23 ear injection model of psoriasiform dermatitis. Our results show that IL-23 supported Treg proliferation and accumulation in inflamed mouse skin. In spite of this fact, IL-23 still drove a significant disease burden suggestive of impaired or insufficient Treg cell responses. In line with this, IL-23 induced the aberrant production of the effector cytokine IL-17A in a fraction of Tregs. Furthermore, these cells shared phenotypic and functional characteristics of prototypic Th17 cells. Not only did these Th17-like Treg cells express RORγt, the master transcription factor of the Th17 lineage, but the development and function of these cells were regulated by RORγt signaling *in vivo*. The generation of this population was also significantly reduced by clinically relevant therapeutics that neutralize TNFα or IL-23. These results show that Th17-like Tregs are generated during inflammation and are modulated by therapeutic modalities that lessen disease severity.

The ontogeny of mouse and human Th17-like Tregs remains a gap in knowledge. This led us to hypothesize that these cells could differentiate from either conventional T cells that up regulate Foxp3 and RORγt or from Tregs that lose lineage conformity to up regulate RORγt and secrete IL-17A^+^ under inflammatory settings. Our results clearly demonstrate that IL-23 drives the generation of Th17-like Tregs preferentially from Treg precursors *in vitro* and we hypothesize that a similar pathway might drive the generation of this population *in vivo*.

In this report, we expand on the observation that in humans a fraction of Tregs in psoriatic skin express IL-17A. We show that IL-23 drives Treg cell plasticity and induces a Th17-like Treg population that is regulated by RORγt signaling. These cells could play a major role in driving and/or exacerbating inflammatory skin diseases like psoriasis. We also define a previously unappreciated role for IL-23 signaling in regulating Treg homeostasis. In addition to expanding our current knowledge on the signaling nodes that regulate Th17-like Tregs, this work could help overcome a significant hurdle in the field to study the plasticity of Th17-like Tregs. Further work to define surface markers for Th17-like Tregs or to develop reporter mouse models to isolate these cells would enable future studies to address whether IL-23 drives lineage instability and plasticity in Tregs to exacerbate disease severity. Such studies would be critical to better understand the role of tissue Tregs in the context of disease and the development of next generation therapeutic modalities.

## Methods

### Mice

Studies were performed with C57BL/6 mice purchased from Charles River Laboratories and housed under specific-pathogen free conditions at AbbVie. Foxp3-GFP reporter mice on the C57BL/6 background were purchased from Jackson Laboratories. Mice used in the studies were between 6–18 weeks of age. All experiments were performed under approval from AbbVie’s Institution Animal Use and Care Committee. All experiments were performed in accordance with the relevant guidelines and regulations.

### Acute intradermal IL-23 ear injection model

Animals received four daily injections of either PBS or recombinant murine IL-23 as previously described^19^. RORγt(i) was dosed at 100 mg/kg (PO) once a day.

### Cell isolation

To isolate cells from ear skin, ears were harvested from euthanized animals and finely minced prior to digestion in 600 µL of RPMI 1640 supplemented with 2 mg/mL Collagenase XI (Sigma), 0.5 mg/mL Hyaluronidase (Sigma) and 0.1 mg/mL DNaseI (Sigma) for 45 mins at 37 °C. Lymph node cells were single cell suspensions of pooled accessory mandibular and superficial parotid lymph nodes.

### Flow cytometry analysis

Single-cell suspensions were stained with a fixable live/dead dye (Invitrogen) prior to Fc receptor blocking and stained for the indicated surface and intracellular proteins. For intracellular cytokine staining, cells were stimulated with phorbol 12-myristate 13-acetate (PMA) (81 nM) and ionomycin (1340 nM) in the presence of brefeldin A (10.6 μM) and monensin (2 μM) (eBioscience) for 3 hours. Intracellular cytokine and transcription factor staining were performed using the Foxp3 transcription factor staining buffer set (eBioscience). Conjugated antibodies were purchased from BD Biosciences, eBioscience or Biolegend. Cells were acquired on a BD FACSAria III or a LSR Fortessa flow cytometer (BD Biosciences) and the data was analyzed using FlowJo (TreeStar).

### *In vitro* differentiation assay

GFP^−^ (Tconv) and GFP^+^ (Treg) CD4^+^ T cells were FACS sorted from Foxp3-GFP reporter mice on a FACSAria Fusion (BD Biosciences). Sorted precursor cells (2 × 10^5^) were stimulated with 2 × 10^5^ Dynabeads Mouse T-Activator CD3/CD28 (ThermoFisher Scientific), 10μg/mL each of anti-IFN-γ (Biolegend) and anti-IL-4 (Biolegend) mAbs in the presence or absence of IL-23 (1μg/mL). Cells were analyzed using the intracellular staining protocol above, 72 hours post stimulation. Cell culture supernatants harvested 72 hours post stimulation was analyzed for IL-17A and IL-10 proteins using cytokine analysis kits (Meso Scale Discovery).

### Statistical analysis

Statistical analysis was performed using GraphPad Prism 6.0 (GraphPad Software). *p* values were calculated using a two-tailed unpaired Student *t* test. A *p* value of < 0.05 was considered significant (*). Error bars represent mean ± standard error of the mean (SEM).

## Supplementary information

Supplementary Information- IL-23 induces regulatory T cell plasticity with implications for inflammatory skin diseases
