# Supplementary material for: IL-23 induces regulatory T cell plasticity with implications for inflammatory skin diseases
Source: Sci Rep. 2019 Nov 27;9:17675. doi: 10.1038/s41598-019-53240-z (PMC6881359; doi:10.1038/s41598-019-53240-z)
Supplement: Supplementary file 1 — Supplementary Information- IL-23 induces regulatory T cell plasticity with implications for inflammatory skin diseases [file 41598_2019_53240_MOESM1_ESM.pdf]

IL-23 induces regulatory T cell plasticity with implications for inflammatory skin diseases

Arun K Kannan[1,*], Zhi Su[1], Donna M Gauvin[3], Stephanie E Paulsboe[1], Ryan Duggan[1], Loren M Lasko[1], Prisca Honore[1], Michael E Kort[1], Steve P McGaraughty[1], Victoria E Scott[1], Stephen B Gauld[1].

[1]Abbvie Inc., 1 North Waukegan Road, North Chicago, IL 60064, USA.

[*]Corresponding author

[3]Former AbbVie employee.

Supplementary Fig. 1

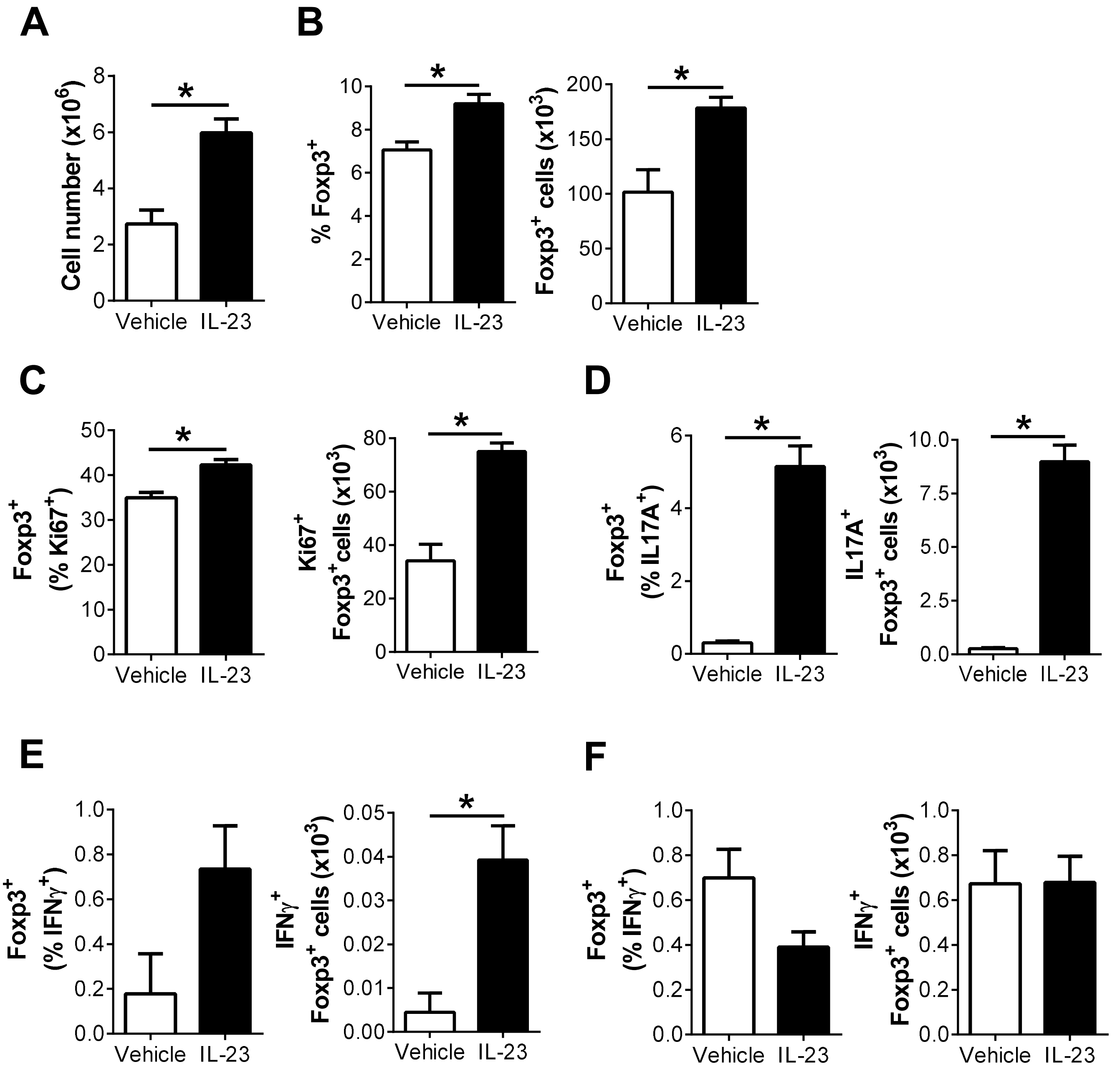

Supplementary Fig. 1.
Mice treated with vehicle or IL-23 analyzed on day 4.
(A) Number of cells in the draining lymph nodes. (B-F) Frequency and number of the indicated cell populations in the ear (E) or draining lymph nodes (B, C, D and F). Pooled data from two independent experiments, n=8. Live $CD45^+CD4^+TCR\beta^+Foxp3^+$ cells in the ear and Live $CD4^+TCR\beta^+Foxp3^+$ cells in the draining lymph nodes are defined as $Foxp3^+$ cells in the figure. *$p<0.05$ using student's t test.

Supplementary Fig. 2

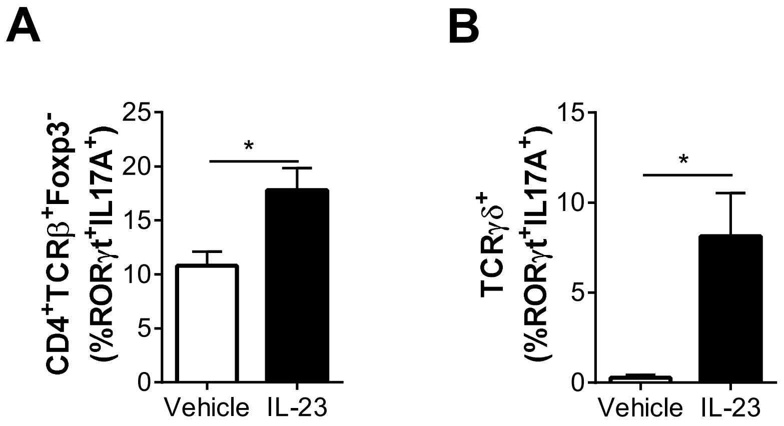

Supporting Fig. 2. Animals received four daily intradermal (ear) injections of vehicle or IL-23 (days 0-3) and were analyzed on day 4. (A and B) Ear skin of mice treated with vehicle or IL-23 was analyzed for the frequency of the indicated T cell populations. All cells were gated on either live $CD45^+$ cells prior to analysis. **p<0.05* determined by student's t test.

Supplementary Fig. 3

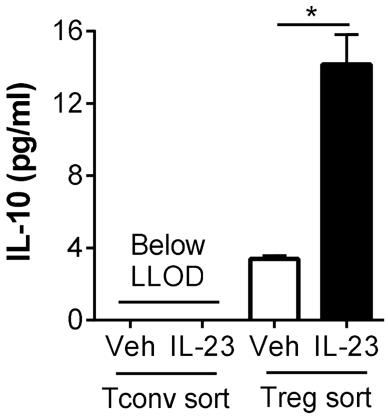

Supplementary Fig. 3
Supernatants of sorted Treg or Tconv cells were treated with vehicle or IL-23 and stimulated with DynaBeads in the presence of αIFN-γ and αIL-4 and analyzed on day 3 for the level of IL-10. Data represents analysis of two independent experiments with cells sorted from pooled spleen and lymph node cells of 4 animals each time. *$p<0.05$ determined by student's t test.
